# Rectosigmoid Endometriosis Mimicking Colorectal Cancer: A Case Report

**DOI:** 10.7759/cureus.105437

**Published:** 2026-03-18

**Authors:** Kawtar Khaissidi, Jihad Karimi, Hajar Ouazzani, Ismail Chaouche, Amal Akammar, Nizar El Bouardi, Badreddine Alami, Meriem Haloua, Karim Ibn Majdoub Hassani, Meryem Boubbou, Mustapha Maaroufi, Moulay Youssef Alaoui Lamrani

**Affiliations:** 1 Radiology Department, Hassan II University Hospital, Fez, MAR; 2 Mother and Child Radiology Department, Hassan II University Hospital, Fez, MAR; 3 General and Visceral Surgery Department, Hassan II University Hospital, Fez, MAR

**Keywords:** bowel obstruction, colorectal cancer mimic, deep infiltrating endometriosis, pelvic magnetic resonance imaging, rectosigmoid endometriosis, tumor-like lesion

## Abstract

Rectosigmoid endometriosis is an uncommon cause of bowel obstruction and may closely mimic colorectal malignancy, particularly when imaging findings are nonspecific. We report the case of a 39-year-old woman who presented with acute bowel obstruction caused by a stenosing rectosigmoid lesion highly suspicious for cancer. Repeated endoscopic biopsies were inconclusive. Surgical management was performed and was complicated by hemorrhagic shock. Final histopathological examination revealed deep infiltrating endometriosis with rectosigmoid involvement. Postoperative imaging demonstrated extensive pelvic involvement of endometriosis, and subsequent attempts at bowel continuity restoration were unsuccessful due to dense adhesions. This case highlights the diagnostic challenges of bowel endometriosis mimicking colorectal cancer and underscores the importance of multidisciplinary evaluation and advanced imaging in women of reproductive age presenting with tumor-like colorectal lesions.

## Introduction

Endometriosis is a chronic estrogen-dependent gynecological disorder defined by the presence of endometrial-like tissue outside the uterine cavity, most commonly affecting women of reproductive age and often associated with pelvic pain and infertility. Endometriosis affects approximately 10% of women of reproductive age [1]. Bowel involvement occurs in nearly 8%-12% of cases [2], with the rectosigmoid colon representing the most frequently affected intestinal segment, accounting for approximately 90% of lesions [3]. In some cases, deep infiltrating endometriosis may present as a pseudo-tumoral lesion, producing clinical and radiologic features that closely mimic colorectal cancer. This diagnostic overlap may lead to delayed diagnosis or unnecessary surgical interventions, particularly in patients presenting with bowel obstruction. We report a case of rectosigmoid endometriosis initially suspected to be colorectal malignancy, highlighting the diagnostic and management challenges associated with this condition.

## Case presentation

A 39-year-old woman presented in February 2018 with no significant medical history and acute bowel obstruction attributed to a suspected rectosigmoid tumor, for which she underwent a diverting ileostomy. The patient had no previously known history of endometriosis, and her gynecological history was otherwise unremarkable.

**Figure 1:**
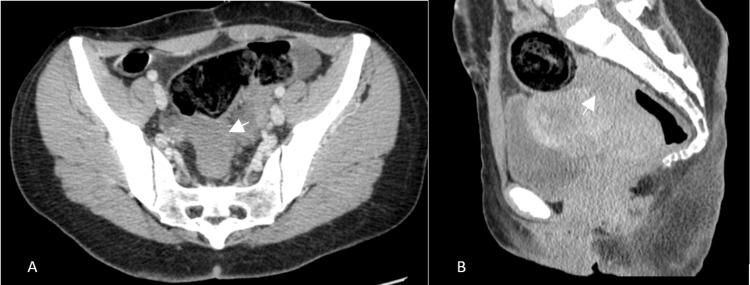
Axial (A) and sagittal (B) pelvic CT images demonstrating a focal area of rectosigmoid wall thickening with transmural contrast enhancement (white arrow), causing luminal stenosis and suggestive of a pseudo-tumoral lesion.

During follow-up, an initial rectoscopy revealed congestive mucosa with nonspecific inflammatory histology. Subsequently, a pelvic CT scan performed in March 2019 demonstrated persistent thickening of the rectosigmoid junction, raising suspicion of a tumoral or pseudo-tumoral process (Figure 1).

Subsequent colonoscopies performed in April 2019 showed a stenosing but passable rectosigmoid lesion, yet repeated biopsies consistently demonstrated only nonspecific subacute colitis without evidence of malignancy. Given the persistent suspicion of a rectosigmoid tumor, the progressive obstructive nature of the lesion, and the lack of diagnostic clarity, surgical management was undertaken in September, 2019. Intraoperatively, the rectosigmoid region was found to be markedly thickened and densely adherent to surrounding pelvic structures, with extensive inflammatory adhesions limiting safe dissection. These anatomical findings precluded complete resection of the lesion. A Hartmann-type procedure with segmental rectosigmoid resection and end colostomy was therefore performed.

The intraoperative course was complicated by significant hemorrhage during pelvic dissection, requiring multiple blood transfusions and vasoactive support. Postoperatively, the patient’s hemodynamic instability was gradually controlled with continued vasopressor therapy and transfusion, allowing stabilization in the intensive care setting. The final histopathological analysis returned in favor of rectosigmoid endometriosis. The patient was subsequently referred to gynecology for specialized management.

**Figure 2:**
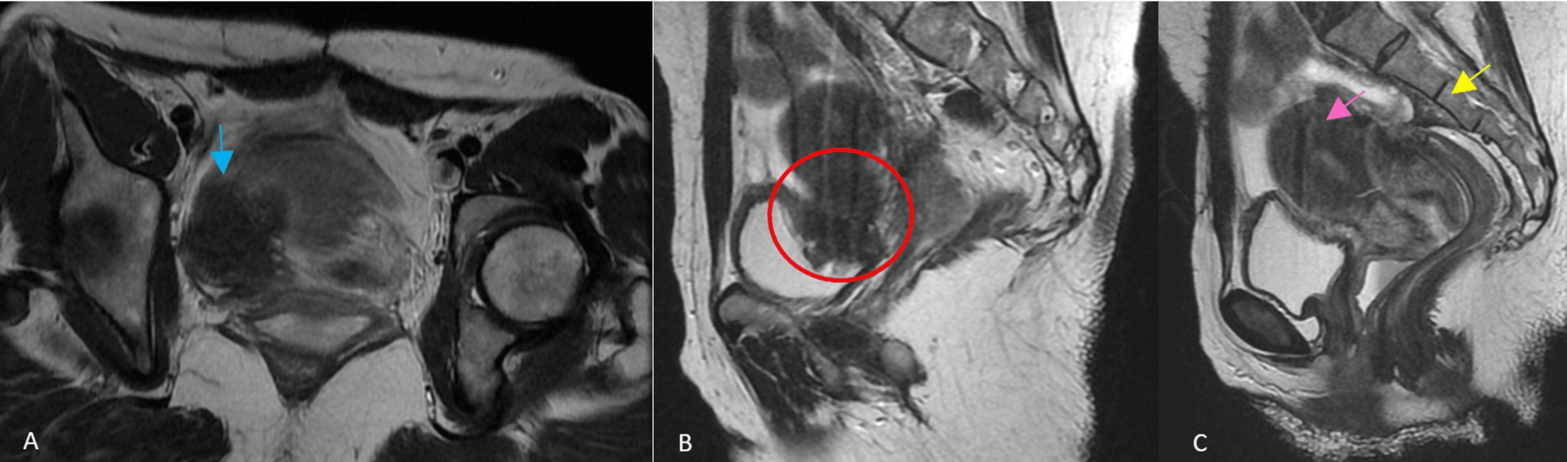
Pelvic T2-weighted MRI images, including an axial view (A) and sagittal views (B and C), demonstrating imaging findings consistent with deep infiltrating endometriosis. The lesions appear as nodular and plaque-like areas of low signal intensity on T2-weighted images, reflecting fibrotic endometriotic implants. Endometriotic lesions involve the urinary bladder (blue arrows) and the vesicouterine pouch (red circle) and extend to the level of the uterine torus (yellow arrow), with associated retraction of adjacent pelvic structures. Additional MRI features suggestive of adenomyosis are also observed, including thickening of the uterine junctional zone (pink arrow).

Obstetric history was unremarkable, and a postoperative pelvic MRI performed in October 2019 demonstrated additional features consistent with deep infiltrating endometriosis, further supporting the diagnosis and guiding ongoing multidisciplinary care (Figure 2).

Three years later, she was re-admitted for planned restoration of bowel continuity; however, extensive adhesions discovered intraoperatively rendered the procedure infeasible. The patient, therefore, continues to live with the stoma and remains under multidisciplinary follow-up involving gynecology, colorectal surgery, and radiology specialists, with clinical monitoring and imaging follow-up to guide further therapeutic decisions.

## Discussion

Endometriosis is a chronic, estrogen-dependent disease characterized by the ectopic growth of endometrial-like tissue, affecting up to 10% of women of reproductive age and frequently associated with pelvic pain and infertility [1]. Deep infiltrating endometriosis represents about 4%-37% of all endometriosis cases [4], and the bowel is involved in 8%-12% of women with endometriosis [2], with the rectosigmoid colon accounting for approximately 90% of cases [3]. Endometriotic lesions can lead to progressive luminal narrowing and a spectrum of cyclic or persistent gastrointestinal symptoms [3]. In advanced stages, they may even result in subacute or complete bowel obstruction, closely imitating colorectal malignancy on both clinical and radiologic evaluations.

Bowel endometriosis predominantly involves the serosa and muscularis propria, with mucosal involvement reported in only 10% of cases [4]. As a result, routine endoscopic biopsies often fail to detect the disease. Our case illustrates this well-described yet underrecognized entity: rectosigmoid endometriosis mimicking a colorectal tumor. As reported in previous studies [5-10], repeated colonoscopies and biopsies in our patient revealed only nonspecific inflammatory changes, despite persistent segmental wall thickening and a stenosing rectosigmoid lesion highly suggestive of malignancy.

In these previously reported cases, as in our patient, the final diagnosis was most often established after surgical resection with histopathological examination of the specimen [5,6,8,9]. In one report, the diagnosis was achieved through cytological analysis combined with histopathology and immunohistochemistry [7], while another rare case revealed malignant transformation of bowel endometriosis into endometrioid adenocarcinoma on the surgical specimen [10].

The differential diagnosis of a rectosigmoid stenosing lesion includes several conditions, particularly colorectal carcinoma, which represents the primary diagnostic concern in this clinical setting. Other potential diagnoses include inflammatory bowel disease, ischemic colitis, infectious colitis, and, less frequently, gastrointestinal stromal tumors or lymphoma. In women of reproductive age, bowel endometriosis should also be considered, especially when imaging demonstrates segmental bowel wall thickening and endoscopic biopsies repeatedly fail to demonstrate malignancy [11].

Cross-sectional imaging plays an important role in the evaluation of rectosigmoid lesions but is not always definitive. CT may demonstrate nonspecific segmental bowel wall thickening or a stenosing mass, as observed in our patient, without pathognomonic features distinguishing deep infiltrating endometriosis from colorectal carcinoma. In contrast, dedicated pelvic imaging, particularly MRI and high-resolution transvaginal or transrectal ultrasound, can more accurately map deep pelvic endometriosis and delineate its relationship with the uterus and surrounding structures. On MRI, deep infiltrating endometriosis typically appears as a hypointense nodular or plaque-like lesion on T2-weighted images due to fibromuscular proliferation, sometimes associated with small hyperintense foci corresponding to ectopic endometrial glands or hemorrhagic components. Conversely, colorectal malignancies more commonly present as irregular masses with asymmetric wall thickening and regional lymphadenopathy [12]. Comparative studies have shown that transvaginal sonography may be slightly superior or at least noninferior to MRI for detecting rectosigmoid deep infiltrating endometriosis, while MRI and 3D rectal ultrasonography provide similar diagnostic accuracy when performed in expert centres [13].

The management of bowel endometriosis requires balancing symptom control, oncologic safety, and the considerable morbidity associated with colorectal surgery. Contemporary literature advocates for an individualized, multidisciplinary approach that brings together gynecologists, colorectal surgeons, radiologists, and pain specialists to optimize outcomes [14]. Segmental bowel resection is generally indicated in cases of marked stenosis, obstructive symptoms, or a high index of suspicion for malignancy, whereas more conservative procedures such as shaving or disc excision may be suitable for selected lesions that do not significantly compromise the bowel lumen [3].

However, these procedures carry a non-negligible risk of postoperative complications, including anastomotic leak, pelvic sepsis, and adhesive disease, and recurrence of endometriosis-related symptoms remains possible even after radical surgery [15]. Long-term morbidity may also include chronic pelvic pain, bowel dysfunction, and complications related to postoperative adhesions, which can significantly impact quality of life and may complicate subsequent surgical procedures. In our case, the patient underwent an extensive colorectal surgical intervention, which resulted in the development of severe intra-abdominal adhesions, ultimately making subsequent restorative surgery technically unfeasible and highlighting the long-term impact of aggressive colorectal surgical management [15]. 

International guidelines increasingly advocate individualized, symptom-directed management of endometriosis, integrating medical therapy and fertility goals with the potential risks of extensive surgery [15]. The significant intraoperative bleeding encountered in our case reflects the technical difficulty of surgery in deep infiltrating endometriosis and further supports the importance of preoperative recognition of the disease. Early diagnosis may allow referral to specialized centres with multidisciplinary surgical expertise.

Hormonal therapies may alleviate pain and reduce lesion activity, but they do not reliably resolve deep bowel nodules and are not sufficient when obstruction or a cancer-like mass is present [3,16]. Our case, together with several reports in the literature [5,6,17-20], supports the recommendation that in women of reproductive age presenting with rectal or rectosigmoid masses and inconclusive endoscopic biopsies, bowel endometriosis should be systematically considered in the differential diagnosis. In this context, early involvement of a multidisciplinary team and the use of advanced pelvic imaging, particularly pelvic MRI, may help better orient the diagnosis, optimize surgical planning, and potentially avoid unnecessary stomas or irreversible colorectal procedures [11].

## Conclusions

Pseudo-tumoral rectosigmoid endometriosis represents a significant diagnostic challenge and may closely mimic colorectal malignancy, sometimes leading to extensive surgical interventions. This case highlights the limitations of standard endoscopic biopsies and underscores the importance of considering bowel endometriosis in women of reproductive age presenting with rectosigmoid stenosis and repeatedly negative biopsies. Dedicated pelvic imaging, particularly MRI, together with multidisciplinary evaluation, may help improve diagnostic orientation and guide management in patients presenting with tumor-like rectosigmoid lesions.
